# Efficacy, safety and therapeutic mechanism of Shen-Qi Xiao-Tan formula in the treatment of peripheral atherosclerosis in patients with type 2 diabetes mellitus: a randomized, double-blind, placebo-controlled trial protocol

**DOI:** 10.1186/s12906-022-03813-9

**Published:** 2022-12-22

**Authors:** Yulin Leng, Xiaoxu Fu, Lisen Qian, Qiqi Li, Hong Gao, Hongyan Xie, Chunguang Xie

**Affiliations:** 1 Hospital of Chengdu University of Traditional Chinese Medicine, No. 39 Shi-Er-Qiao Road, Chengdu, Sichuan Province People’s Republic of China; 2 Traditional Chinese Medicine Regulating Metabolic Diseases Key Laboratory of Sichuan Province, 39 Shi-Er-Qiao Road, Chengdu, Sichuan Province People’s Republic of China

**Keywords:** Type 2 diabetes mellitus, Peripheral atherosclerosis, Chinese herbal medicine, Randomized controlled trial, Shen-Qi Xiao-Tan formula

## Abstract

**Background:**

Peripheral atherosclerosis is a common macrovascular complication of diabetes, but the treatment is limited. Chinese herbal medicine is the complementary and alternative therapy to delay the progression of atherosclerosis and reduce blood glucose and lipids. Shen-Qi Xiao-Tan (SQXT) formula is one of the prescriptions commonly used to treat diabetic peripheral atherosclerosis, but there is still a lack of high-quality evidence-based evidence.

**Methods:**

This is a randomized, double-blind, placebo-controlled add-on trial that is expected to enroll 114 diabetic patients with peripheral atherosclerosis. After a 2-week run-in period, participants will been randomly assigned in a 1:1 ratio and receive 12 weeks of usual treatment and SQXT formula (treatment group) or usual treatment and placebo (control group). The primary outcome is the change in carotid intima-media thickness from baseline to endpoint. The secondary outcomes are the structure and function of peripheral arteries, blood glucose and lipids, traditional Chinese medicine syndrome score, and quality of life, and safety and endpoint events are evaluated. To explore the therapeutic mechanism through oxidative stress, inflammation, and advanced glycation end products, and lipidomics will be used to screen for biomarkers for diagnosis and efficacy evaluation.

**Discussion:**

The objective of this trial is to evaluate the efficacy, safety and therapeutic mechanism of SQXT formula in the treatment of diabetic peripheral atherosclerosis. It will obtain high-quality evidence-based evidence and promote the treatment of diabetic macroangiopathy and the research and development of new drugs.

**Trial registration:**

This trial is registered on Chinese Clinical Trials.gov with number ChiCTR2100047189 on 10 Jun 2021, and has been approved by the Ethical Review Committee of Hospital of Chengdu University of Traditional Chinese Medicine with number 2020KL-080.

## Background

Diabetes, as one of the major chronic diseases that threaten human health, has become a world-wide public health problem. According to the International Diabetes Federation Diabetes Atlas (10th edition), there are about 537 million people living with diabetes worldwide [1]. It estimates that total diabetes-related health expenditure will reach USD 1.03 trillion by 2030 [1]. Diabetes and its complications have brought heavy medical and economic burdens to individuals and society.

Macroangiopathy is the main cause of death and disability in diabetes, with atherosclerosis as the basic pathological alteration. Compared with non-diabetic patients, type 2 diabetic patients with atherosclerosis have the characteristics of earlier onset, more severe degree, and wider range of disease [2–5]. In China, the total number of diabetic patients is about 140.9 million, ranking first in the world [1]. The China DIA-LEAD study shows that the prevalence of macroangiopathy in diabetic patients is as high as 55.6% in China, and peripheral vascular disease is the most common one, with the prevalence of about 31.4% [2]. Atheroscleroses of the carotid and lower extremity arteries are one of the most common causes of peripheral artery disease. Carotid artery atherosclerosis can further cause head and neck artery stenosis, atherosclerotic stroke, transient ischemic attack, myocardial infarction, etc. [6]. Lower extremity arteries atherosclerosis can further causes lower extremity artery disease, diabetic foot ulcers, limb gangrene, amputation, and it is an important risk factor for cardiovascular events [7–9]. Globally, an adult dies from diabetes and its complications every 8 s, and a patient is lost to lower extremity amputation every 30 s as a consequence of diabetes [1, 10, 11]. Approximately 75% of diabetics die from macroangiopathy [12, 13]. A series of complications caused by peripheral atherosclerosis have caused the increase of readmission rates and length of stay, the raise of medical costs, and the consumption of a large number of medical and public health resources [14, 15]. Therefore, delaying peripheral atherosclerosis progression as soon as possible to reduce the occurrence of fatal and disabling events is the key content of diabetes management.

At present, the clinical prevention and control measures for diabetic macroangiopathy include strict blood glucose control, anti-hypertensive agents, lipid lowering therapies, anti-platelet agents, etc. But the comprehensive clinical control rate is only 8.1% [2]. A number of large clinical trials have shown that strict blood glucose control is associated with increased risk for death in diabetic patients at high risk of cardiovascular events [16–19]. The Look AHEAD Research found that intensive lifestyle intervention was associated with increased risk for primary cardiovascular outcome in patients with poor glucose control (HR 1.85, 95%CI 1.32–2.61) [20]. How to comprehensively relieve diabetic peripheral atherosclerosis is a challenging clinical issue.

In China, Chinese herbal medicine (CHM) is widely used to treat diabetic macrovascular complications. A systematic review and meta-analysis has shown that CHM can reduce blood glucose and haemoglobin A1c (HbA1c), ameliorate lipid metabolism disorders, and improve Traditional Chinese Medicine (TCM) syndrome scores in the treatment of diabetic macrovascular disease [21]. Shen-Qi series formulas have efficacy for alleviating insulin resistance, promoting b-cell function, and improving intestinal microbiota in the treatment of patients with type 2 diabetes mellitus (T2DM) [22, 23].In addition, it can reduce blood interleukin-6 (IL-6), hs-C-reactive protein and carotid intima-media thickness (CIMT), alleviate clinical symptoms, and improve quality of life in diabetic patients with macroangiopathy [24, 25]. Furthermore, the mechanism of Shen-Qi series formulas in delaying the progression of diabetic atherosclerosis has been proved from epigenetic DNA methylation, PI3K-Akt pathway, glucagon-like peptide-1 and other aspects [26–28]. A systematic review and meta-analysis has shown that the efficacy of Shen-Qi series formulas combined with usual treatment is superior to that of usual treatment in the treatment of type 2 diabetic macrovascular complications, it can reduce fasting and postprandial blood glucose, C-reactive protein and leptin, and improve adiponectin [29]. On the other hand, it has found that there are deficiency in previous clinical trials, such as lack of allocation concealment and blinding methods, and small sample size, which led to limited quality of trials.

In summary, CHM can regulate the overall pathological status of diabetic patients with macroangiopathy through multi-target, but there is a lack of high-quality clinical trials. This trial will comprehensively evaluate the efficacy, safety and therapeutic mechanism of Shen-Qi Xiao-Tan (SQXT) formula in the treatment of peripheral atherosclerosis in T2DM from the structure and function of peripheral arteries, cardiovascular events, lipid metabolism, inflammation, oxidative stress and advanced glycation end products (AGEs).

### Objective

#### Primary objective

The primary objective of this trial is to determine whether SQXT formula is superior to placebo to relieve peripheral atherosclerosis in T2DM by evaluating the following outcomes.

1. Change in CIMT.
2. Changes in the structure and function of peripheral arteries, including (1) hyperechoic thickness of the arterial lumen wall, vascular internal diameter, peak systolic velocity (PSV), end diastolic velocity (EDV), mean velocity (Vm), pulse index (PI) and resistance index (RI) of lower extremity and carotid arteries; (2) flow-mediated vasodilation (FMD); (3) ankle brachial index (ABI).
3. Levels of glucose and lipid profile indices.
4. TCM syndrome score.
5. Quality of life as measured by multiple questionnaires.

#### Secondary objectives

This trial also aims to evaluate safety and explore therapeutic mechanism of SQXT formula in treating patients with diabetic peripheral atherosclerosis.

## Methods/design

### Study design

**Fig. 1:**
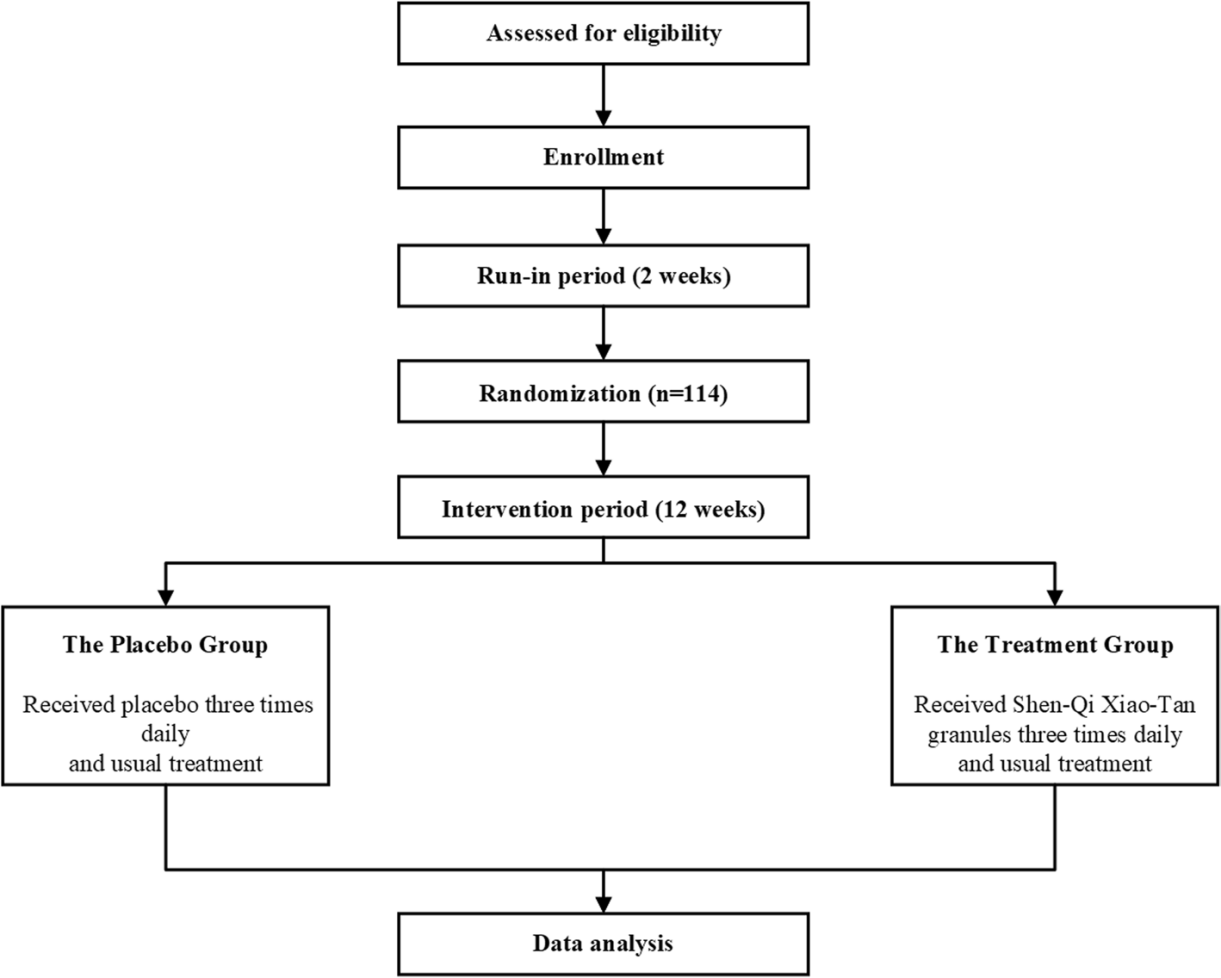
The flow chart of the trial

This trial is registered on Chinese Clinical Trials.gov with number ChiCTR2100047189 on 10 Jun 2021. It is a randomized, double-blind, placebo-controlled, add-on trial. 114 subjects are expected to be enrolled. After a 2-week’s run-in period, participants who meet the standards of the run-in period are randomly assigned to 12 weeks of either placebo group or treatment group on a 1:1 allocation. The flow chart of the trial is shown in Fig. 1. This protocol follows the requirements of the *Standard Protocol Items for Randomized Trials* (SPIRIT) and the *Consolidated Standards of Reporting Trials* (CONSORT) Extension for CHM Formula [30, 31].

### Inclusion criteria

1. According to *Standards of Medical Care in Diabetes*, *Guideline for the Prevention and Treatment of T2DM in China*, and *Guideline for Ultrasound Examination of Blood Vessels and Superficial Organs*, [32–34]
  patients with definite diagnosis of T2DM;
  color doppler ultrasound of the carotid or lower extremity arteries showed intima-media thickening (IMT ≥ 1 mm) and/or atherosclerotic plaque (limited IMT ≥ 1.5 mm).
2. Patients with the following symptoms of TCM syndromes: thirst, fatigue, body fat, dietary predilection of fat and sweets, etc. According to *Guiding Principles for New Drug Clinical Research of TCM* and *Guidelines for Prevention and Treatment of Diabetes in TCM*, the diagnostic criteria of deficiency of both qi and yin with phlegm-turbidity syndrome are defined on the standards and expert advice [35, 36].
3. Aged between 30 and 65 years old.
4. Patients participate voluntarily and sign the Informed Consent Form (ICF).
5. Women of childbearing age must have a negative urine pregnancy test and agree to use reliable contraception during this trial.

### Exclusion criteria

1. Patients with calcified plaques or unstable plaques in lower extremity or carotid arteries, arterial stenosis (vascular inner diameter stenosis ≥ 50%), or arterial occlusion.
2. Patients who have been diagnosed with coronary heart disease by coronary angiography, or whose electrocardiogram and clinical manifestations suggest coronary heart disease, or who have had cardiovascular and cerebrovascular events, such as acute coronary syndrome and cerebral infarction.
3. ABI ≤ 0.9.
4. Fasting TG > 5.7 mmol/L.
5. Patients with abnormal liver and kidney function, such as the ratio of serum alanine aminotransferase (ALT) to aspartate aminotransferase (AST) 1.5 times higher than the upper limit of normal value of that, creatinine higher than the upper limit of normal value of that, estimated GFR ≤ 60 ml·min^−1^·(1.73m^2^)^−1^.
6. Patients with acute metabolic disorders such as diabetic ketoacidosis, or severe stress, occurred within 1 month before screening.
7. Patients with serious illnesses whose life expectancy does not exceed 2 years, or malignant tumor, or mental diseases.
8. Patients who are known to be allergic to the ingredients in SQXT formula or its preparations.
9. Having a history of alcoholism or drug abuse.
10. Women who are preparing for pregnancy, or pregnant, or lactating women.

### Recruitment

Participants will be recruited from Hospital of Chengdu University of TCM. This is also China National Clinical Research Base of TCM for diabetes.

### Randomization

The random sequence was generated by the third-party statistician from the Sichuan Province Evidence-based Medicine Center of TCM using R software (version 4.0.3), including 114 random numbers in a 1:1 ratio between the treatment group and the control group. It was concealed by opaque sealed envelopes.

### Blinding

Investigators, participants, outcome assessors, and data analysts will be blinded. Outcome assessors will follow the standard operating procedure to complete tests. The third-party drug management staff from the Sichuan Province Evidence-based Medicine Center of TCM will blind investigational drugs according to random sequence, so that the drugs could not be distinguished according to their numbers. The drug management staff shall not directly participate.

The data analysts will be unblinded at the first level after the database is locked, and unblinded at the second level after statistical analysis is completed. In case of emergency such as serious adverse event and suspected unexpected serious adverse reaction, the participant’s allocated intervention will be revealed urgently, and investigator should treat he and record the time, date and reason of unblinding.

In order to ensure stability of the drugs, increase convenience of taking medicine, and remove the influence factors such as the method of decoction and the origin of medicinal materials, SQXT formula and placebo will be used in granules form. In accordance with the *Standard for the Use of Food Additives in the National Standard for Food Safety*, the placebo is made from maltodextrin and food additives such as food colorants and bitters. SQXT formula and placebo are similar in color, smell, taste, appearance, packaging, and tag. Both of them are produced by Sichuan Neo-Green Pharmaceutical Technology Development Co. Ltd. (Sichuan, China).

### Interventions

#### Run-in period

In order to standardize the usual treatment, obtain the baseline values of blood glucose, blood lipid and blood pressure (BP), and ensure the compliance of participants, participants will enter a 2-week’s run-in period after passing the screening.

According to the recommendations of multidisciplinary guidelines, the investigators will decide the usual treatment based on the participants' personal situation [32, 33]. All participants will receive the same lifestyle behavior management education in diabetes self-management, diet, and routine physical activity. All of them will be treated with standard treatment of glucose-lowering medications and antihypertensive medications, as well as atorvastatin to lower lipid.

Participants should not use other CHM, acupuncture, Tuina and other TCM interventions outside of this trial, as well as drugs with potential risk of cardiovascular disease events. In consideration of clinical practice and ethical principles, drugs with demonstrated cardiovascular disease benefits will not prohibited in this trial.

Participants will be assigned randomly to the intervention period only if their blood glucose, blood lipid and BP reach the following targets [32, 33, 37, 38]: (1) fasting plasma glucose (FPG) between 4.4 and 7.0 mmol/L; (2) non-fasting blood glucose < 10.0 mmol/L; (3) BP ≤ 140 / 90 mmHg; and (4) low-density lipoprotein cholesterol (LDL-C) < 3.4 mmol/L.

#### Intervention period

Treatment group: Participants will take usual treatment and SQXT formula granules, which is composed of Shengshaishen (Dried fresh Ginseng), Zhihuangqi (Astragalus membranaceus), Shanyao (Common Yam Rhizome), Shengdihuang (Unprocessed Rehmannia Root), Niuxi (Twotoothed Achyranthes Root), Fabanxia (Processed Pinellia Tuber), Zexie (Oriental Waterplantain Rhizome), Mugua (Common Floweringqince Fruit) and Danshen (Danshen Root). The action of each herb is summarized in Table 1. The dosage of SQXT formula is 3 times a day, one bag (15 g per bag) at a time. Participants will dissolve the granules in hot water (100 ml), then stir and cover for 3 to 5 min, then take it orally between 30℃ to 36℃ (ante cibum).

**Table 1:** The action of each Traditional Chinese Medicinal herb

PinYin name	English name	Latin name	Action / Pharmaceutical study
Shengshaishen	Dried fresh Ginseng	*Radix Ginseng*	1. Enhancing immune function; 2. Anti-thrombosis effect; 3. Inhibition of oxidative stress
Zhihuangqi	Astragalus membranaceus	*Radix Astragali seu Hedysari*	1. Enhancing immune function; 2. Promoting metabolic effect; 3. Lipid-lowering effect; 4. Inhibition of platelet aggregation; 5. Anti-thrombosis effect
Shanyao	Common Yam Rhizome	*Rhizoma Dioscoreae*	1. Enhancing immune function; 2. Lipid-lowering effect; 3. Glucose-lowering effect; 4. Inhibition of oxidative stress
Shengdihuang	Unprocessed Rehmannia Root	*Radix Rehmanniae Recens*	1. Bidirectional regulatory effect on immune; 2. Anti-inflammatory effect; 3. Shortening of blood clotting time; 4. Cardiovascular system protecting effect
Niuxi	Twotoothed Achyranthes Root	*Radix Achyranthis Bidentatae*	1. Enhancing immune function; 2. Anti-inflammatory effect; 3. Analgesic effect
Fabanxia	Processed Pinellia Tuber	*Rhizoma Pinelliae Preparatum*	1. Anti-atherosclerosis effect; 2. Inhibition of oxidative stress; 3. Antihypertensive effect; 4. Anti-aging effect
Zexie	Oriental Waterplantain Rhizome	*Rhizoma Alismatis*	1. Lipid-lowering effect; 2. Inhibition of oxidative stress; 3. Anti-inflammatory effect; 4. Anti-atherosclerosis effect; 5. Diuresis effect
Mugua	Common Floweringqince Fruit	*Fructus Chaenomelis*	1. Enhancing immune function; 2. Hepatoprotective effect; 3. Anti-inflammatory effect
Danshen	Danshen Root	*Radix Salviae Miltiorrhizae*	1. Inhibition of platelet aggregation; 2. Anti-atherosclerosis effect; 3. Lipid-lowering effect; 4. Inhibition of oxidative stress

Control group: Participants will take usual treatment and placebo granules. The specifications and usage of placebo are exactly the same as SQXT formula.

#### Outcome measurement

Changes of the following outcomes from baseline (week 0) to endpoint (week 12) of intervention will be evaluated.

#### Primary efficacy outcome

CIMT [39].

#### Secondary efficacy outcomes

1. Hyperechoic thickness of the arterial lumen wall, vascular internal diameter, PSV, EDV and Vm of bilateral carotid arteries (common carotid and internal carotid artery) and lower extremity arteries (femoral artery, anterior tibial artery, posterior tibial artery and dorsalis pedis artery), and PI, RI and S/D will be calculated according to the formula: PI = (PSV-EDV) / Vm; RI = (PSV-EDV) / PSV; S/D = PSV / EDV.
2. FMD of the brachial artery.
3. ABI.
4. FPG and HbA1c.
5. Total cholesterol (TC), triglycerides (TG), high density lipoprotein cholesterol (HDL-C) and LDL-C.
6. TCM syndrome score [35].
7. The indices for evaluating the quality of life are as follows: (1) The MOS 36-item short-form health survey (SF-36); (2) Quality of life scale for patients with T2DM (DMQLS); (3) Chinese version of diabetes management self-efficacy scale (C-DMSES).

#### Endpoint outcomes

The incidence of endpoint events and the time to progression to endpoint will be evaluated. Endpoints events include:

1. The progression of peripheral artery disease is defined as bilateral carotid and lower extremity arteries stenosis or arterial occlusion.
2. Major adverse cardiac events include cardiovascular death, non-fatal myocardial infarction, ischemic stroke, and hospitalization for heart failure.
3. Diabetic foot ulcer.

#### Safety outcomes

Adverse event (AE) will be collected after the participants begin to receive drugs. The record of AE includes the following: descriptive analysis, time of occurrence and termination, severity, frequency of occurrence, whether treatment is needed, and if necessary, the treatment given needs recording.

Participants will be monitored for vital signs (body temperature, respiration, heart rate, BP) during the outpatient visit every 4 weeks, and will be asked for if they have any symptoms of discomfort.

Participants will have the following tests at the beginning and end of intervention: blood, urine and stool routine examination, electrocardiogram, liver function test (ALT, AST, TBIL, AKP, GGT), and renal function test (blood urea nitrogen, creatinine). Systemic symptoms of participants will be closely observed. If a participant has suspicious symptoms of respiratory system, circulatory system, liver or kidney disease, he will be immediately tested on safety.

#### Therapeutic mechanism outcomes

1. Tumor necrosis factor-α (TNF-α), IL-6.
2. Reactive oxygen species (ROS), superoxide dismutase (SOD).
3. AGEs.
4. Lipidomics.

### Sample size

This is a randomized, double-blind, placebo-controlled superiority trial. The primary outcome indicator, CIMT, is used as the basis for sample size estimation. The mean of CIMT of the placebo group is 1.12 mm [40–42]. In this trial, the mean of CIMT of the treatment group is estimated at 1.03 mm, and the standard deviation of two groups is 0.10. The superiority margin is 0.03 (one-sided test, α = 0.025, 1-β = 0.8). The sample allocation ratio between the two groups is 1:1. Using PASS 15 software, the minimum sample size is calculated to be 90. In addition, considering the dropout rate of 20%, we plan to enroll a total of 114 participants.

### Data collection

The basic data are as follows: (1) demographic including gender, age, height, weight, waist circumference, hip circumference, body mass index and waist-to-hip ratio; (2) vital signs; (3) duration of diabetes; (4) other medical history and treatment history; (5) women of childbearing age should have a urine pregnancy test, and be inquired at each visit to record their menstrual history.

Blood and urine samples should be collected in the morning with an empty stomach for at least 8 h. The fasting serum samples for detection of TNF-α, IL-6, ROS, SOD and AGEs will be stored at -20 °C. It will be detected with enzyme-linked immunosorbent assay by an independent laboratory from JUJIN BIOTECHNOLOGY CORPORATION (Chengdu, China). The fasting plasma samples for detection of lipidomics will be stored at -80 °C. It will be detected with non-targeted lipidomics analysis platform based on Q Exactive (Thermo Scientific™) mass spectrometry system by Shanghai Lu Ming Biotech Co., Ltd. (Shanghai, China). Other outcomes will be measured by Hospital of Chengdu University of TCM on the same day.

### Drop-out criteria

1. Participants who have intolerable adverse reaction and withdraw voluntarily during the trial.
2. The ratio of ALT to AST of participants is 3 times higher than the upper limit of normal value of that, and it is still abnormal after review within a week.
3. Creatinine of participants is 1.5 times higher than the upper limit of normal value of that, and it is still abnormal after review within a week.
4. Participants who fail to take the drugs as required or have incomplete data.
5. Participants violate the protocol to change or add drugs that are not within the prescribed scope and affect the assessment of efficacy.
6. Participants who are lost to follow-up without explanation.
7. Participant who is unblinding.

They will be required to return for the last observation as much as possible, and the relevant data will be included in the datasets.

### Termination criteria

This trial will be terminated under the following criteria: (1) clustered serious adverse events are related to intervention measurement with supportive evidence; (2) the administration requested that this trial be discontinued.

### Data management

The original data included ICF, CaseReport Form (CRF), record form of distribution and withdrawal of drug, medication administration monitoring form and medication self-assessment form.

Paper CRF will be used to record data, and EpiData software (version 3.1) will be used for data management. Developing a data verification plan to set up logic verification. Consistency testing will be carried out through double data entry, and the inconsistent results will be checked and corrected against CRF item by item until they are completely consistent. The data administrator will screen the questions according to the manual verification plan in the data verification plan.

Finally, the electronic database will be locked after the project leader, data analyst and data administrator sign the database locking record, then will be submitted to the data analyst.

### Data analysis

The statistical analysis will be conducted by the third-party statistician from the Sichuan Province Evidence-based Medicine Center of TCM using Stata software (version 16.0). The statistical analysis plan is completed and confirmed before the start of trial.

The Full Analysis Set (FAS) includes participants who have been randomized to receive the drugs at least once. Missing data will be used the last observation carried forward (LOCF) method. The Per-protocol set (PPS) includes participants who follow the protocol to implement the inclusion and exclusion criteria and interventions, and complete all the visit plans. It does not include participants who drop-off, lost to follow-up, and discontinue medication for any reason. Only participant with an effective medication rate between 80 and 120% can be included in PPS analysis. The safety set (SS) includes participants who have been randomized to receive the drugs at least once and have experienced AE.

The descriptive statistics of enumeration data will be presented as frequency and percentage. Normally distributed variables will be presented as mean and standard deviation, and non-normally distributed variables will expressed by median and interquartile range. Difference between groups will be assessed with t-test, analysis of variance (ANOVA), rank sum test, and chi-square (χ2) test. Survival analysis will be used for comparison of endpoint outcome between groups, and Kaplan–Meier curve will be used to explore each possible relevant factor. The cox multiple regression model will be used to detect the influence of prognostic factors on endpoint outcome if the amount of data allows. AE will be analyzed and described in detail, and the χ2 test will be used to compare the differences between groups if necessary. The statistical significance is defined as *p* ≤ 0.05.

Subgroup analysis will be based on HbA1c. Level of HbA1c will be stratified into ≥ 8.0% and < 8.0% to evaluate correlation between glycemic control of diabetic patients with macroangiopathy and outcome indicators [37].

## Discussion

Thickening of CIMT is an early sign of atherosclerosis and a recognized risk factor for arteriosclerotic cardiovascular disease. A systematic review and meta-analysis showed that each 0.1 mm increase in CIMT is associated with a 15% increase in the risk of coronary heart disease and a 17% increase in the risk of stroke [43]. It is recommended as an early assessment of peripheral atherosclerosis in patients with T2DM [44, 45]. Because diabetic peripheral atherosclerosis is a chronic disease, it is difficult to observe sufficient endpoint events in short-term studies. Hence, CIMT is often used as the objective surrogate indicator for primary efficacy outcome or endpoint outcome in clinical studies and systematic reviews of diabetic macrovascular complications [39, 46]. Therefore, CIMT is used as the primary efficacy outcome in this trial.

Color doppler ultrasound has unique value in the qualitative and positioning diagnosis of vascular lesions, which can evaluate the structure of arterial lumen, the nature of atherosclerotic plaque and the hemodynamics. ABI test is a good tool to evaluate the degree of stenosis and obstruction of lower extremity arteries, with high sensitivity and specificity [47, 48]. Brachial artery FMD can evaluate nitric oxide releasing function of endothelial cells to reflect the vascular endothelial function [49]. Vascular endothelial cells regulate the balance between vasodilation and contraction, anti-inflammatory and pro-inflammatory, anti-oxidation and pro-oxidation, and release vasoactive substances to maintain vascular tone and structure [50–52]. Vascular endothelial dysfunction is an early warning of cardiovascular disease [53]. Therefore, FMD test plays an important role in early recognition and effective intervention of vascular disease progression [54]. The above tests have the advantages of convenience, non-invasiveness, and repeatable measurement. They are used to objectively evaluate the changes in structure and function of peripheral arteries.

This trial will evaluate short-term efficacy from the vascular structure and function of carotid and lower extremity arteries, glucose and lipid level, and quality of life, and long-term benefits from cardiovascular and lower extremity vascular events, and explore whether the effectiveness of the drug is related to improve inflammation, oxidative stress, and accumulation of AGEs. Moreover, lipidomics will be used to screen the biomarkers for this disease diagnosis and treatment. Lipid metabolism disorders are involved in the onset and progression of diabetes and atherosclerosis. Lipidomics provides a systematic quantitative and qualitative analysis of lipids in vivo and reveals the regulation of lipid metabolism [55]. However, the limitation of this trial is that the elderly (> 65 years) will not be included in order to exclude the influence of aging on vascular damage within a limited sample size, which leads to insufficient evidence for extrapolation of the trial results to the elderly population.

## Trial status

The recruitment of participants has started in June 2021 and is going.

## Data Availability

Not applicable. Details of the current study are available from the corresponding author. The study results will be presented in medical journals or academic conferences, and will be disseminated among doctors and patients.

## Abbreviations

ABI: Ankle brachial index
AE: Adverse event
AGEs: Advanced glycation end products
ALT: Alanine aminotransferase
AST: Aspartate aminotransferase
BP: Blood pressure
CHM: Chinese herbal medicine
CIMT: Carotid intima-media thickness
CRF: Case Report Form
EDV: End diastolic velocity
FMD: Flow-mediated vasodilation
FPG: Fasting plasma glucose
HbA1c: Haemoglobin A1c
HDL-C: High density lipoprotein cholesterol
ICF: Informed Consent Form
IL-6: Interleukin-6
LDL-C: Low-density lipoprotein cholesterol
PI: Pulse index
PSV: Peak systolic velocity
RI: Resistance index
ROS: Reactive oxygen species
SOD: Superoxide dismutase
SPIRIT: Standard Protocol Items for Randomized Trials
SQXT: Shen-Qi Xiao-Tan
T2DM: Type 2 diabetes mellitus
TC: Total cholesterol
TCM: Traditional Chinese Medicine
TG: Triglycerides
TNF-α: Tumor necrosis factor-α
Vm: Mean velocity

## References

[1] International Diabetes Federation. IDF Diabetes atlas 10th edition 2021. https://diabetesatlas.org/atlas/tenth-edition/. Accessed 13 Sep 2022.

[2] Zhang X, Ran X, Xu Z, Cheng Z, Shen F, Yu Y (2018). Epidemiological characteristics of lower extremity arterial disease in Chinese diabetes patients at high risk: a prospective, multicenter, cross-sectional study. J Diabetes Complications.

[3] Xu Y, Wang L, He J, Bi Y, Li M, Wang T (2013). Prevalence and control of diabetes in Chinese adults. JAMA.

[4] Rao KondapallySeshasai S, Kaptoge S, Thompson A, Di Angelantonio E, Gao P, Sarwar N (2011). Diabetes mellitus, fasting glucose, and risk of cause-specific death. N Engl J Med.

[5] Xu Y, Bi Y, Li M, Wang T, Sun K, Xu M (2013). Significant coronary stenosis in asymptomatic Chinese with different glycemic status. Diabetes Care.

[6] Salonen JT, Salonen R (1991). Ultrasonographically assessed carotid morphology and the risk of coronary heart disease. Arterioscler Thromb.

[7] Bragg F, Holmes MV, Iona A, Guo Y, Du H, Chen Y (2017). Association between diabetes and cause-specific mortality in rural and urban areas of China. JAMA.

[8] Bommer C, Sagalova V, Heesemann E, Manne-Goehler J, Atun R, Bärnighausen T (2018). Global economic burden of diabetes in adults: projections from 2015 to 2030. Diabetes Care.

[9] Mitka M (2007). Report quantifies diabetes complications. JAMA.

[10] Bakker K, Apelqvist J, Lipsky BA, Van Netten JJ, International Working Group on the Diabetic Foot (2016). The 2015 IWGDF guidance documents on prevention and management of foot problems in diabetes: development of an evidence-based global consensus. Diabetes Metab Res Rev..

[11] Amoah VMK, Anokye R, Acheampong E, Dadson HR, Osei M, Nadutey A (2018). The experiences of people with diabetes-related lower limb amputation at the Komfo Anokye Teaching Hospital (KATH) in Ghana. BMC Res Notes.

[12] Gao R (2012). Evolution and future perspectives for cardiovascular interventions in China. EuroIntervention.

[13] Gao R (2010). Current status of percutaneous coronary intervention in China. Heart.

[14] Rice JB, Desai U, Cummings AK, Birnbaum HG, Skornicki M, Parsons NB (2014). Burden of diabetic foot ulcers for medicare and private insurers. Diabetes Care.

[15] Raghav A, Khan ZA, Labala RK, Ahmad J, Noor S, Mishra BK (2018). Financial burden of diabetic foot ulcers to world: a progressive topic to discuss always. Ther Adv Endocrinol Metab.

[16] Turnbull FM, Abraira C, Anderson RJ, Byington RP, Chalmers JP, Control Group (2009). Intensive glucose control and macrovascular outcomes in type 2 diabetes. Diabetologia.

[17] Gerstein HC, Miller ME, Byington RP, Goff DC, Bigger JT, Action to Control Cardiovascular Risk in Diabetes Study Group (2008). Effects of intensive glucose lowering in type 2 diabetes. N Engl J Med.

[18] Duckworth W, Abraira C, Moritz T, Reda D, Emanuele N, Reaven PD (2009). Glucose control and vascular complications in veterans with type 2 diabetes. N Engl J Med.

[19] Gaede P, Lund-Andersen H, Parving HH, Pedersen O (2008). Effect of a multifactorial intervention on mortality in type 2 diabetes. N Engl J Med.

[20] Bancks MP, Chen H, Balasubramanyam A, Bertoni AG, Espeland MA, Kahn SE (2021). Type 2 diabetes subgroups, risk for complications, and differential effects due to an intensive lifestyle intervention. Diabetes Care.

[21] Guo Y, Shi Y (2017). Clinical Effect of traditional chinese medicine compound for treating diabetes mellitus and its effect on blood glucose and blood lipid. Chin Arch Tradit Chin Med.

[22] Zhang X, Liu Y, Xiong D, Xie C (2015). Insulin combined with Chinese medicine improves glycemic outcome through multiple pathways in patients with type 2 diabetes mellitus. J Diabetes Investig.

[23] Zhang XX, Liu WF, Xiong R, Yang BT, Zhong M, Liu XH (2019). Effect of shenqi compound recipe on intestinal microecology of patients with qi and yin deficiency and blood stasis syndrome newly diagnosed type 2 diabetes mellitus. Chin J Exp Tradit Med Formulae.

[24] Fu XX, Xie CG (2013). Clinical observation on modified shenqi compound for 57 cases of macrovascular disease in type 2 diabetes. J Tradit Chin Med.

[25] Xie HY, Xie CG, Gao H (2014). Effect of traditional chinese medicine compound on Hs-CRP and IL6 of patients with vascular lesions in type 2 diabetes. Chin J Exp Tradit Med Formulae.

[26] Gao H, Duan Y, Fu X, Xie H, Liu Y, Yuan H (2018). Comparison of efficacy of SHENQI compound and rosiglitazone in the treatment of diabetic vasculopathy analyzing multi-factor mediated disease-causing modules. PLoS One.

[27] Fu XX, Gao H, Liu Y, Xie HY, Yuan HP, Xie CG (2019). The bidirectional regulation of Shenqi Compound on vascular endothelial growth in diabetic macrovascular disease rats is studied based on expression profile microarray. Liaoning Journal of Traditional Chinese Medicine.

[28] Qin HZ, Guo BG, Fu XX, Li HY, Chao J, Fang CM (2017). Research on the protecting function of vascular endothelium by shenqi compound formula through regulating the expression of differential genes. China Journal of Traditional Chinese Medicine and Pharmacy.

[29] Liao TT, Deng LY, Xiao YP, Deng YP, Tu X, Xie CG (2018). Systematic evaluation of randomized controlled study on shenqi compound in the treatment of type 2 diabetes mellitus with vascular lesions. Pharmacology and Clinics of Chinese Materia Medica.

[30] Chan AW, Tetzlaff JM, Gøtzsche PC, Altman DG, Mann H, Berlin JA (2013). SPIRIT 2013 explanation and elaboration: guidance for protocols of clinical trials. BMJ.

[31] Cheng CW, Wu TX, Shang HC, Li YP, Altman DG, Moher D (2017). CONSORT Extension for Chinese Herbal Medicine Formulas 2017: Recommendations, Explanation, and Elaboration (Simplified Chinese Version). Ann Intern Med.

[32] American Diabetes Association. 10. Cardiovascular Disease and Risk Management: Standards of Medical Care in Diabetes-2021. Diabetes Care. 2021;44(Suppl 1):S125-S150. 10.2337/dc21-S010. 10.2337/dc21-S010 33298421

[33] Chinese Diabetes Society. Guideline for the prevention and treatment of type 2 diabetes mellitus in China (2020 edition). Chin J Diabetes Mellitus 2021;13(04): 317–411.

[34] Ultrasound Society of Chinese Medical Doctor Association (2011). Guideline for Ultrasound Examination of Blood Vessels and Superficial Organs.

[35] Zheng XY (2002). Guiding principles for new drug clinical research of traditional Chinese medicine (Trial).

[36] Tong XL, Liu XM, Wei JP, Ni Q, Gao QJ (2011). Guidelines for Prevention and Treatment of Diabetes in TCM. Chinese Medicine Modern Distance Education of China.

[37] American Diabetes Association. 6. Glycemic Targets: Standards of Medical Care in Diabetes-2021. Diabetes Care. 2021;44(Suppl 1):S73-S84. 10.2337/dc21-S006. 10.2337/dc21-S006 33298417

[38] China Joint Committee on Revision of Guidelines for Prevention and Treatment of Dyslipidemia in Adult (2016). Guidelines for prevention and Treatment of Adult Dyslipidemia in China (Revised edition, 2016). Chinese Circulation J.

[39] Vascular Medicine Committee of China Medical Education Association, Vascular Professional Group, Beijing Cardiology Society, Chinese Medical Association, Community Prevention and Treatment Center for Vascular Diseases of Peking University Health Science Center (2018). Guidelines for the Application of Vascular Health Assessment System in China (the 3rd report in 2018). Nat Med J China.

[40] Mita T, Katakami N, Shiraiwa T, Yoshii H, Onuma T, Kuribayashi N (2016). Sitagliptin Attenuates the Progression of Carotid Intima-Media Thickening in Insulin-Treated Patients With Type 2 Diabetes: The Sitagliptin Preventive Study of Intima-Media Thickness Evaluation (SPIKE): A Randomized Controlled Trial. Diabetes Care.

[41] Crouse JR, Raichlen JS, Riley WA, Evans GW, Palmer MK, O'Leary DH (2007). Effect of rosuvastatin on progression of carotid intima-media thickness in low-risk individuals with subclinical atherosclerosis: the METEOR Trial. JAMA.

[42] Talari HR, Zakizade M, Soleimani A, Bahmani F, Ghaderi A, Mirhosseini N (2019). Effects of magnesium supplementation on carotid intima-media thickness and metabolic profiles in diabetic haemodialysis patients: a randomised, double-blind, placebo-controlled trial. Br J Nutr.

[43] van den Oord SC, Sijbrands EJ, ten Kate GL, van Klaveren D, van Domburg RT, van der Steen AF (2013). Carotid intima-media thickness for cardiovascular risk assessment: systematic review and meta-analysis. Atherosclerosis.

[44] Huang Y, Bi Y, Wang W, Xu M, Xu Y, Li M (2011). Glycated hemoglobin A1c, fasting plasma glucose, and two-hour postchallenge plasma glucose levels in relation to carotid intima-media thickness in chinese with normal glucose tolerance. J Clin Endocrinol Metab.

[45] Meschia JF, Bushnell C, Boden-Albala B, Braun LT, Bravata DM, Chaturvedi S (2014). Guidelines for the primary prevention of stroke: a statement for healthcare professionals from the American Heart Association/American Stroke Association. Stroke.

[46] Nathan DM, Lachin J, Cleary P, Orchard T, Brillon DJ, Backlund JY (2003). Intensive diabetes therapy and carotid intima-media thickness in type 1 diabetes mellitus. N Engl J Med.

[47] Pearson TA (2002). New tools for coronary risk assessment: what are their advantages and limitations?. Circulation.

[48] Suzuki E, Kashiwagi A, Nishio Y, Egawa K, Shimizu S, Maegawa H (2001). Increased arterial wall stiffness limits flow volume in the lower extremities in type 2 diabetic patients. Diabetes Care.

[49] Soga J, Noma K, Hata T, Hidaka T, Fujii Y, Idei N (2011). Rho-associated kinase activity, endothelial function, and cardiovascular risk factors. Arterioscler Thromb Vasc Biol.

[50] Radke RM, Diller GP, Duck M, Orwat S, Hartmann D, Thum T (2014). Endothelial function in contemporary patients with repaired coarctation of aorta. Heart.

[51] Zheng H, Patel M, Hryniewicz K, Katz SD (2006). Association of extended work shifts, vascular function, and inflammatory markers in internal medicine residents: a randomized crossover trial. JAMA.

[52] Toff WD, Jones CI, Ford I, Pearse RJ, Watson HG, Watt SJ (2006). Effect of hypobaric hypoxia, simulating conditions during long-haul air travel, on coagulation, fibrinolysis, platelet function, and endothelial activation. JAMA.

[53] Maruhashi T, Soga J, Fujimura N, Idei N, Mikami S, Iwamoto Y (2013). Relationship between flow-mediated vasodilation and cardiovascular risk factors in a large community-based study. Heart.

[54] Shechter M, Shechter A, Koren-Morag N, Feinberg MS, Hiersch L (2014). Usefulness of brachial artery flow-mediated dilation to predict long-term cardiovascular events in subjects without heart disease. Am J Cardiol.

[55] Wenk MR (2005). The emerging field of lipidomics. Nat Rev Drug Discov.

